# Transfusion Medicine in the year 2025: Facts or Fantasy?

**DOI:** 10.4103/0973-6247.39501

**Published:** 2008-01

**Authors:** N. Choudhury

**Affiliations:** *Medical Director, Prathama Blood Center, Vasna, Ahmedabad - 380 007, Gujarat, India*

Blood transfusion science has made tremendous progress over the last century. It started with the discovery of ABO blood groups by Karl Landsteiner at the beginning of the century. The journey continued throughout the century towards safer blood transfusion. This ‘march’ was in the direction of achieving zero risk transfusion. Initial focus was targeted towards gathering knowledge about different aspects of red cell antigen structures. As a result, major blood group antigens were discovered around the first half of the last century. Red cell serology made an opening to the new horizon called immunohematology. Research scope gradually extended to other cellular components. Towards the end of the century, all cellular blood components were brought to the use of human beings in one way or the other. Most interesting developments were in the field of progenitor cells.

Few months back, in one evening, I was resting on the terrace of my house, all alone, after a long day of hard work, trying to recollect what transpired during the day. Gradually, I drifted into thinking what will happen in the coming years to blood banking and the field of transfusion medicine. In that week, I discussed with a few friends to consolidate my thoughts about ‘Transfusion Medicine by the Year 2025’.

The first thought that came to mind was whether this specialty would survive at all beyond 2025. Transfusion therapy is life-saving; however, it is need-based. If we look into the need, it differs across different specialties and between specialties; the need varies according to the individual's clinical situations. Transfusion need depends upon multiple factors. It has been observed that blood requirement has come down in many surgical procedures. It is because of improved techniques, use of better equipment and expertise gained by surgeons and clinicians. Good examples are open heart surgery procedures and gastro surgery procedures like cholecystectomy. About a decade back, it was a routine practice to use (or keep in reserve) two to four units of blood for these types of procedures. However, blood utilization has come down drastically for the above reasons. Moreover, transfusion services are spreading awareness to avoid unnecessary transfusions and to use blood judiciously.

Emergence of new specialties is another hallmark of medical sciences in the previous century. This emergence was due to high-end medical care and segregation of specialties into super-specialties. A good example is stem cell therapy, which has become a routine mode of treatment in many countries. Because of high-end medical interventions, the demand for blood and blood components is increasing gradually all over the world. The demand for blood is gradually increasing although some branches of medicine have brought down its necessity.

In the field of blood banking, the main focus of attention remains in making transfusion therapy safe. Scientists involved in this field are making concerted efforts to improve methodologies and techniques. The journey begins with very simple tests like microscopy or passive haemagglutination to highly sensitive tests like nucleic acid test. Efforts were made for higher sensitivity of diagnostic tests to increase the detectable limit of pathogens. Apart from increasing the sensitivity of different tests, modern tests were included as newer pathogens were continuously emerging as threats. Some pathogens were never thought to be transfusion-associated threats. Due to change in the behavior of human and animals, improved knowledge and detection techniques as well as better vigilance in the part of transfusion services, new diseases associated with transfusion have come to limelight.

In the last century, transfusion medicine had become a semi-clinical discipline. Unlike other pre- and para-clinical specialties, it dealt not only with patient's samples but also with blood donors and patients who are alive. For the blood donor, blood bankers carry out simple procedures like screening and whole blood collection to complex procedures like apheresis, stem cell harvesting, cord blood banking, etc. On the other hand, this specialty has gone to the bedside, as we call it ‘bed side transfusion medicine’. There is active involvement in patient consultation by clinicians. Many centers are even taking up complex procedures like therapeutic apheresis and exchange procedures in patients as bedside procedures.

In this article, we have summed up what we have been seeing in practical life or reading the literature. What will be the future of transfusion medicine? What progress will be made? Will this specialty survive beyond 2025? We do not know! I discussed with few specialists in this field and tried to provoke their thinking for the future of transfusion medicine. Their reactions about the status of this specialty by the year 2025 were vivid. I will try to pen our thoughts together in following paragraphs. None of us knew what would become reality.

There is anxiety in the minds of blood bankers, , ‘What would happen if substitutes for blood components come in the market?’ This is a genuine fear due to the advancement of research at the molecular level. Quite a few commercial companies are working hard to find out substitutes for red blood cells (RBC). One of these almost succeeded in developing a hemoglobin solution; however, the whole project was scrapped and millions of dollars dissipated. To come up with a substitute for RBC is a Herculean task due to the complex nature and function of the cell. Not much of information in literature or the internet is available on this subject because of the involvement of patents and secret nature of research carried out by (mainly) commercial companies. Recently, advancement was made in the development of universal red cells by using enzymes and by blocking antigen sites on the RBC surface. This will make RBC transfusion safer by removing and reducing antigenicity of the cells. However, a question remains about feasibility of producing this component at commercial level and its price. In case of the artificial oxygen-carrying substances, we may see it in practical use by the year 2025. Some research materials may come into use at the level of clinical trials but it is unlikely they will come to the market. In the most optimistic tone, if anything equivalent to RBC may come to the market, it cannot probably be able to replace RBC because of price and biological half-life. Artificial blood will be helpful for immediate transfusion in case of resuscitation. However, it seems that blood transfusion services are going to survive for another 15 or 20 years (?) because patients with chronic requirements always need their help. Moreover, cost will be a prohibitive factor if artificial blood comes into reality. Especially in developing countries, blood transfusion will continue for years to come with more focus on safety.

Another future prospect is to develop artificial blood from culture plate. Genetically modified blood cells are targeted for production in laboratory. Although it sounds a fantasy, progress can be expected within 10 or 15 years. Advantages in these types of blood components will be no risk of transfusion, neutral blood group and no fear of alloimmunization. However, the success and wide use of these components is questionable because of anticipated high price and risk of development in artificial culture medium.

Transfusion medicine specialists from many developed and developing countries are already involved in the different phases of stem cell collection, processing, preservation and transfusion. Stem cell culture and manipulation has already been taken up by many blood centers in developed countries. The role of stem cells in different areas of medical sciences is gradually increasing, especially in R&D. Recently, clinicians and scientists from different fields of medical sciences have started using stem cells for different purposes, including infusing in cardiac, spinal, neural tissues and other areas in the human body. This is just a beginning. Transfusion medicine specialists have good prospects in the field of R&D in stem cell biology.

There will be much progress in protein chemistry. At present, most of the therapeutic proteins for clinical use come from fractionation. There is much advancement in protein production by recombinant technology. Another cutting-edge technology in the horizon will be transgenic technology where high value and purified proteins will be produced for infusion/transfusion. Genetically modified female animals will produce human coagulation factors (e.g. F.VIII) and proteins in the milk as engineered by scientists. The limitations of this technology at present are low volume of production and high cost involvement.

There would be more involvement from the side of central/regional blood centers in tissue banking activities. Bone banking, skin banking and sperm banking may become a routine banking facility in the major blood centers. Associated facilities like donor and patient screening for HLA, TTI testing and post-transfusion/transplant monitoring will be an integral part of the blood centers’ responsibilities. Cryobiology will be another new frontier opened to transfusion medicine specialists. Manipulation and preservation of different liquid and solid tissues using cryobiology, documentation, retrieval and supply will be a routine job for these specialists. Blood bankers are most suitable for this job because they are very familiar with production, testing, preservation, regulatory affairs and documentations.

It is hoped that some major changes may take place in the blood banking service sector in developing countries like India. There will be restructuring of blood transfusion services for better efficiency and service delivery. The number of blood banks will come down, and high-capacity, technically up-scaled central blood banks will function in their place to reduce the costs of operation. Two types of blood transfusion services would exist in future. One will be the large central/regional blood centers as mentioned earlier. There will be smaller hospital-based blood banks having responsibilities limited only to that hospital. They may not collect blood units but receive tested blood units from the central/regional blood centers for use by the hospital. Transfusion medicine specialists placed in these blood banks will focus more on the clinical interface including clinical consultations and bedside transfusion medicine. They will explore into other areas of interest including tissue banking. Excessive control and interference by the managerial personnel in the daily working of technical personnel is not advisable because blood banking in the hands of administrators usually becomes another mode of profit-making.

As a whole, we foresee good prospects for transfusion medicine specialists by the year 2025, at least in the developing countries. However, traditional blood banking will gradually fade out. Transfusion medicine specialists will lead big blood centers. They will be more clinically oriented and actively associated with transfusion regime in the patients. There will be opening for at least one specialist in every big hospital. Stem cell production and dispensing will be a routine job. In such competitive environment, ‘survival of the fittest’ is the benchmark. Blood transfusion services - both central blood banks and hospital blood banks – will place highly skilled and qualified specialists.

